# Distribution of β-Lactamase Genes in Clinical Isolates from California Central Valley Hospital Deviates from the United States Nationwide Trends

**DOI:** 10.3390/antibiotics10050498

**Published:** 2021-04-27

**Authors:** Candace Guzman-Cole, Fabian Santiago, Sona Garsevanyan, Suzanne Sindi, Miriam Barlow

**Affiliations:** 1 Department of Molecular and Cell Biology, School of Natural Sciences, University of California Merced, Merced, CA 95343, USA; ccole5@ucmerced.edu (C.G.-C.); sgarsevanyan@ucmerced.edu (S.G.); 2 Department of Applied Mathematics, School of Natural Sciences, University of California, Merced, CA 95343, USA; fsantiago3@ucmerced.edu (F.S.); ssindi@ucmerced.edu (S.S.)

**Keywords:** ESBL, selection, Enterobacteriaceae, antibiotic resistance, stewardship

## Abstract

The evolution and dissemination of antibiotic resistance genes throughout the world are clearly affected by the selection and migration of resistant bacteria. However, the relative contributions of selection and migration at a local scale have not been fully explored. We sought to identify which of these factors has the strongest effect through comparisons of antibiotic resistance gene abundance between a distinct location and its surroundings over an extended period of six years. In this work, we used two repositories of extended spectrum β-lactamase (ESBL)-producing isolates collected since 2013 from patients at Dignity Health Mercy Medical Center (DHMMC) in Merced, California, USA, and a nationwide database compiled from clinical isolate genomes reported by the National Center for Biotechnology Information (NCBI) since 2013. We analyzed the stability of average resistance gene frequencies over the years since collection of these clinical isolates began for each repository. We then compared the frequencies of resistance genes in the DHMMC collection with the averages of the nationwide frequencies. We found DHMMC gene frequencies are stable over time and differ significantly from nationwide frequencies throughout the period of time we examined. Our results suggest that local selective pressures are a more important influence on the population structure of resistance genes in bacterial populations than migration. This, in turn, indicates the potential for antibiotic resistance to be controlled at a regional level, making it easier to limit the spread through local stewardship.

## 1. Introduction

β-lactam antibiotics have been in use since the discovery of penicillin in the 1940s, and they continue to be the most widely used antibiotics due to their high effectiveness, ease of delivery, and low toxicity [1,2]. The longstanding use of β-lactam antibiotics has led to the emergence of resistant strains in clinical care settings [3]. The continuous selection and evolution of β-lactamase genes by β-lactam antibiotic use has led to the diversification of successful β-lactamase genes: *bla*_TEM_, *bla*_SHV_, *bla*_CTX-M_, and *bla*_OXA_ [4]. β-lactamase genes produce extended spectrum β-lactamase (ESBL) enzymes that work by hydrolyzing β-lactam antibiotics, rendering them ineffective. *bla*_TEM_ and *bla*_SHV_ were the first β-lactamase enzymes identified in 1963 and 1972, respectively, and were implicit in outbreaks in the 1990s [5,6,7]. Today, *bla*_SHV_ composes 10% of ESBLs, and *bla*_TEM_ has become somewhat less common in the U.S. [8]. *bla*_CTX-M_ was first identified in 1989, and was identified with increasing frequency throughout the 1990s [9]. By the 2000s, the frequency of *bla*_CTX-M_ enzymes surpassed those of *bla*_TEM_ and *bla*_SHV_. Although first discovered in 1976, *bla*_OXA_ enzymes have been increasing in prevalence due to the frequent association of *bla*_OXA-1_ with *bla*_CTX-M-15_ [10,11]. Today, *bla*_CTX-M_ enzymes are the most identified ESBLs, and have displaced *bla*_TEM_ and *bla*_SHV_ in many individual hospitals [6,9,12,13,14]. However, this trend is not uniform across publications originating from different surveillance locations [11,15,16]. Bajpai et al. (2017) found *bla*_TEM_ to be the most abundant ESBL enzyme in a single hospital, although other reports detail different ESBL gene frequencies [17]. In the United States, few recent nationwide surveillance studies have specifically examined the frequencies of specific ESBL genes. One recent survey of 26 hospitals identified *bla*_TEM_ as the most abundant ESBL enzyme in clinical isolates (47%), followed by *bla*_CTX-M_ (36%), *bla*_SHV_ (35%), and *bla*_OXA_ (20%) [18].

Regional variance in the frequencies of ESBLs enables the assessment of which factors are contributing the most to ESBL frequencies. Due to the strong selection that bacteria experience from antibiotics and the rapid migration of bacteria that occurs in human populations, selection and migration were the two factors we chose to investigate. To understand the relative contributions of selection and migration, it was important to obtain and compare updated ESBL gene frequencies. We chose to compare the frequencies of ESBLs in a local repository of ESBL positive isolates collected from a single hospital, with average frequencies nationwide across the U.S. obtained from ESBL-positive clinical isolates whose genomic sequences have been deposited in the NCBI Isolates Browser Database.

When comparing genetic variations over two populations, there are four possible outcomes depending on the stability of gene frequencies within a site and comparisons of those frequencies between sites. First, gene frequencies that are stable over time within a population and non-uniform across populations indicate low migration between bacterial populations and that selection for resistance within a given population is strong and constant. However, if the gene frequencies are unstable over time within a population and non-uniform across populations, this would indicate alternating local selective pressures and rapid migration as “immigrants would increase the mutation supply rate” [19] and would compete with “better-adapted residents maintaining the population away from the local fitness optimum” [20]. Stability over time and uniformity between populations suggest rapid continuous migration between populations and strong consistent selection resulting in a highly resistant and optimized strain [20]. Finally, unstable (alternating) frequencies over time and uniformity between populations indicate strong alternating selective pressures in large areas (or populations). Moreover, this signal also indicates rapid migration because variation between populations averages out as immigration leads to a decrease in genetic differentiation between populations [21]. We compared ESBL gene frequencies from Dignity Health Mercy Medical Center (DHMMC) and the rest of the U.S. over a period of six years as follows.

## 2. Results

### 2.1. Regional Gene Frequencies

**Figure 1:**
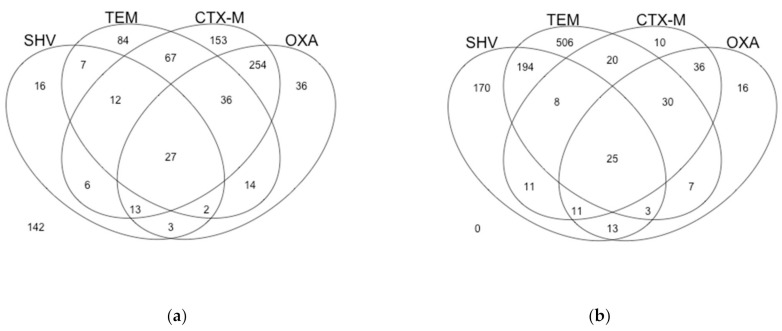
Venn diagrams of *bla*_SHV_, *bla*_TEM_, *bla*_CTX-M_, and *bla*_OXA_ combinations from both repositories. (**a**) Venn diagram of the resistance genes found in the clinical isolates from DHMMC. There were 142 isolates without any of these resistance genes (inconclusive data). (**b**) Venn diagram of the resistance genes found in the nationwide database of ESBL clinical isolates.

We performed a molecular surveillance study of common β-lactamases among isolates. At DHMMC, the most common ESBL gene we identified was *bla*_CTX-M_, followed by *bla*_OXA_, *bla*_TEM_, and *bla*_SHV_ (Figure 1a). Their yearly frequencies are provided in Table 1. Mathematical analysis of those frequencies over time revealed no significant differences over months or agricultural seasons. However, there were some significant differences (*p*-value < 0.05) in yearly frequencies (Table 1). *bla*_SHV_ and *bla*_TEM_ frequencies were stable over time. *bla*_CTX-M_ frequencies increased after the first year in 2014 and remained stable over time (Table S3). *bla*_OXA_ frequencies significantly decreased in 2016 from previous years but returned to stable in 2017 (Table S4).

We then measured the frequencies at which these genes co-occurred in each isolate population. Statistical analysis suggests a genetic linkage (or correlation) between resistance genes in isolates from DHMMC (Table 2). Two statistical methods (Pearson’s chi-square test and the phi coefficient) revealed a significant positive correlation between *bla*_TEM_ and *bla*_SHV_ (*p*-value < 0.05) and between *bla*_CTX-M_ and *bla*_OXA_ (*p*-value < 0.05). There are significant negative correlations between *bla*_TEM_ and *bla*_CTX-M_ (*p*-value < 0.05) and *bla*_TEM_ and *bla*_OXA_ (*p*-value < 0.05). 

When stratified by species, the resistance genes in *E. coli* and *K. pneumoniae* isolates from the DHMMC clinical isolates show unique correlations (Table 2). In *E. coli* isolates, *bla*_CTX-M_ and *bla*_OXA_ are positively correlated with one another (*p*-value < 0.05) and negatively correlated with *bla*_TEM_ (*p*-value < 0.05). However, in *K. pneumoniae*, all the resistance genes are positively correlated with each other (*p*-value < 0.05) but significance is lost for *bla*_TEM_ and *bla*_OXA_ after the false discovery rate (FDR)-controlling procedure. 

### 2.2. U.S. Database Gene Frequencies

We conducted an analogous surveillance study using a nationwide database of ESBL clinical isolates from the NIH Pathogen Detection Isolates Browser (Figure 1b). Nationwide, the resistance gene frequencies were different from the DHMMC repository. The most common ESBL gene was *bl*a_TEM_, followed by *bla*_SHV_, *bla*_CTX-M_, and *bla*_OXA_. Those frequencies are provided in Table 3. Analysis of these gene frequencies over time also showed no significant differences in the frequencies of common ESBL genes in the U.S. over a period of months. However, there were significant differences (*p*-value < 0.05) in the yearly frequencies for *bla*_SHV_, *bla*_TEM_, and *bla*_CTX-M_ (Table 3). *bla*_SHV_ significantly increased in 2015, followed by a significant decrease in 2016, but returned to stable in 2017 (Table S5). *bla*_TEM_ frequencies significantly decreased in 2017 and 2018 from previous years (Table S6). There was a non-significant decrease in *bla*_CTX-M_ frequency in 2015 from previous years. The 2015 *bla*_CTX-M_ frequency was significantly different than that of 2018 due to an increase that year (Table S7).

In the nationwide database, the co-occurrence of resistance genes differs from that of our local samples from DHMMC (Table 4). We observed a negative association between *bla*_TEM_ and the other resistance markers (*p*-value < 0.05), and a positive association between *bla*_CTX-M_ and *bla*_OXA_ (*p*-value < 0.05). The negative association of *bla*_TEM_ with both *bla*_CTX-M_ and *bla*_OXA_ was almost double that found at DHMMC for all species (Table 2 and Table 4). When stratified by species, *E. coli* upholds these trends, but in *K. pneumoniae*, *bla*_SHV_ is negatively correlated with the other resistance genes (*p*-value < 0.05) and *bla*_CTX-M_ is positively correlated *bla*_OXA_ (*p*-value < 0.05).

### 2.3. Comparison of DHMMC and U.S. Populations

We then performed a formal statistical comparison between the frequencies of resistance genes in the DHMMC repository and the U.S. database to determine significant differences (Table 5). In the nationwide database, *bla*_SHV_ and *bla*_TEM_ occur more frequently (all *p*-values < 0.05) than at DHMMC. At DHMMC, *bla*_CTX-M_ and *bla*_OXA_ occur at higher frequencies than they do nationwide (all *p*-values < 0.05). A further breakdown of the gene frequencies by species and FDR controlling revealed no significant frequency difference in *bla*_SHV_ within species between datasets. *bla*_TEM_ occurs in 57–68% more *E. coli* isolates in the U.S. database than *E. coli* isolates at DHMMC (*p*-values < 0.05). There is no significant difference in bla_TEM_ frequency in *K. pneumoniae* isolates. *bla*_CTX-M_ occurs much more frequently in *E. coli* and *K. pneumoniae* isolates from DHMMC than in the U.S. database (all *p*-values < 0.05). *bla*_CTX-M_ occurs in 47–58% more *E. coli* isolates and 33–52% more *K. pneumoniae* isolates at DHMMC than isolates from the nationwide U.S. database. This trend is similar but less drastic for *bla*_OXA_, which occurs in 26–36% more *E. coli* isolates and 26–44% more *K. pneumoniae* isolates than isolates nationwide.

## 3. Discussion

We answered our initial question about the relative importance of selection and migration in small and large regions. The stability of resistance genes over time in a distinct community that differ from the nationwide frequencies strongly suggests that local selective pressures have a larger impact on frequencies than migration. DHMMC is unique with regards to the presence of *bla*_CTX-M_ and *bla*_TEM_ genes. At DHMMC, *bla*_CTX-M_ occurs more frequently than in the nationwide database, while *bla*_TEM_ occurs less frequently at DHMMC. The negative correlation at DHMMC between *bla*_TEM_ and *bla*_CTX-M_ and between *bla*_TEM_ and *bla*_OXA_ implies incompatibilities between *bla*_TEM_ and at least one of the other genes. Since *bla*_CTX-M_ and *bla*_OXA_ are commonly linked with each other, it is not surprising that *bla*_TEM_ is negatively associated with both of them, and genetic incompatibilities may exist for only one of those pairings. As those genetic compatibilities were not observed throughout the U.S., it is likely that they are the product of local selective pressures. This negative relationship results mainly from *E. coli* isolates; this relationship is not observed in *K. pneumoniae* isolates. This result indicates that either the genetic background of *K. pneumoniae* eliminates the incompatibilities of these genes, or that the antibiotic exposures of these pathogens is different from *E. coli.* Additionally, at DHMMC, there appears to be strong selection for *bla*_CTX-M_, which may be displacing *bla*_TEM_ likely due to antagonism between these genes [22].

In terms of antimicrobial stewardship, our results suggest resistance may be modulated at a regional level, facilitating the implementation of effective strategies to limit and control selection of the antibiotic resistance genes. However, we also found evidence that resistance must be modulated differently for separate species, which may be difficult to conduct because of the environmental presence of antibiotics and the use of empiric therapies without species identification.

High *bla*_SHV_ frequencies are unique to our nationwide dataset. However, when we considered *E. coli* and *K. pneumoniae* isolates separately, DHMMC and nationwide frequencies of *bla*_SHV_ are similar. The greater number of *K. pneumoniae* isolates in the nationwide database likely accounts for the overall higher nationwide frequencies of *bla*_SHV_ (Simpson’s paradox [23]). A high *bla*_TEM_ frequency is also unique to our nationwide dataset. There are 57–68% more *E. coli* isolates with *bla*_TEM_ nationwide than isolates from DHMMC. *E. coli* isolates nationwide have a negative correlation between *bla*_TEM_ and the other resistance genes, meaning these isolates are likely to have *bla*_TEM_ and no other resistance genes. In *K. pneumoniae* isolates nationwide, there is a negative correlation between *bla*_SHV_ and the other resistance genes, meaning these isolates are likely to have *bla*_SHV_ and no other resistance genes, which is the opposite of the *K. pneumoniae* isolates from DHMMC. ESBLs were initially derived from *bla*_SHV_ and *bla*_TEM_, explaining the relatively high *bla*_TEM_ frequencies nationwide, as *bla*_TEM_ has proceeded to fixation [24]. *bla*_SHV_ and *bla*_TEM_ have been responsible for most ESBL infections since at least the 1980s, so it is reasonable for them to be widely distributed in a large-scale dataset [25].

We observed stable gene frequencies over time within the DHMMC population which differ from nationwide frequencies. This implies there is low migration or low survival of immigrants between populations, and that the selective forces within the DHMMC population are strong and constant. Overall, there is strong evidence that local selective pressures have a much stronger effect on the frequencies of ESBLs in local populations. This suggests that communities and specific regions have the potential to effectively manage ESBL frequencies through intentional antibiotic stewardship practices.

## 4. Materials and Methods

### 4.1. Hospital Isolates

Clinical patient isolates (*n* = 872) were collected from patients at DHMMC in Merced, CA, USA, from 2013 to 2018. These isolates were flagged as ESBL using Vitek 2 (bioMérieux, Inc. Hazelwood, MO, USA). These patient samples were collected from urine, blood, sputum, and wounds.

### 4.2. Molecular Methods

Genomic DNA was isolated using the boil preparation method by adding a single colony to 100 µL sterile water and boiling at 100 °C for 15 min. From this 100 µL solution, 1 µL was used in the PCR reaction for the respective genes with the primers listed in Table 6. Multiplex PCR was used to determine the presence of *bla*_CTX-M_, *bla*_TEM_, and *bla*_OXA_. Detection of *bla*_SHV_ was run in a separate reaction. Each PCR reaction consisted of 1 µL of template DNA, 10 µM of each primer, and Taq 2X master mix (NEB) at a final concentration of 1X, and the reactions were run under the following conditions: initial denaturation at 94 °C for 10 min, 30 cycles of 94 °C for 40 s, 60 °C for 40 s, 72 °C for 1 min, and a final elongation at 72 °C for 7 min [26]. PCR amplicons were run out on 2% agarose gel at 100 V for 30 min and visualized using a ChemiDoc™ Touch Imaging System. (Figures S1 and S2).

### 4.3. U.S. Database

We obtained clinical isolate genomes from the NCBI RefSeq database [27], using the NIH Isolate Browser [28] to identify clinical isolates of *E. coli* and *K. pneumoniae* from the United States from 2013 to 2018. Using the Comprehensive Antibiotic Resistance Database (CARD) [29], we identified isolate genomes containing ESBL genes to compile an ESBL clinical database (*n* = 1060) using the BLAST+ program. In combination with a 98% identity cut-off to positively identify the frequency of *bla*_TEM_, *bla*_OXA_, *bla*_CTX-M_, and *bla*_SHV_, we applied an additional base pair match cutoff for each gene to limit partial gene matches. For *bla*_TEM_, we required a base pair (bp) match at or above 753 bp; for *bla*_OXA_, we required 831 bp; for *bla*_CTX-M,_ we required 876 bp; and for *bla*_SHV_, we required 861 bp. The metadata for nationwide clinical isolate genomes were downloaded from the NIH Isolate Browser and included date, species, and location. The list of genome assemblies used to perform this analysis can be found in the Supplementary Materials.

### 4.4. Statistical Analysis

We used one-way analysis of variance (ANOVA) to compare the means of the resistance gene frequencies to identify significant differences between frequencies across months. We compared the same months across years, different months within the same year, different months across all years, and bins of 2, 3, 4, and 6 months. We tested for significant differences between the means of the resistance gene frequencies across years at DHMMC and the nationwide database using a Z-test [30]. We tested for significant differences in the proportions of a resistance marker between isolates from DHMMC and the nationwide database using a Z-test. Pairwise linkage among the resistance alleles in each of the two clinical isolate populations, DHMMC and the nationwide database, was assessed using a chi-square test [31]. The phi coefficient (PC) was used as a chi-square measure of directional deviation from the null relationship of independent assortment [32]. The PC has the desired property of accounting for sample size (often >500 in this work) and because it has a known sampling distribution, it allows us to compute significance and to form confidence intervals. Controlling for multiple statistical tests was conducted via the FDR-controlling procedure [33], a Bonferroni-type multiple testing procedure, with a false discovery control level of q* = 0.025. Only those results that remained significant after using FDR are reported as significant. All analyses were performed using the Statistics and Machine Learning Toolbox of MATLAB R2020a [34].

## Figures and Tables

**Table 1:** The yearly frequency of *bla*_SHV_, *bla*_TEM_, *bla*_CTX-M_, and *bla*_OXA_ from DHMMC. Each frequency is presented with a 95% confidence interval. The number of isolates is given in the first column in parenthesis.

DHMMC	*bla*_SHV_	*bla*_TEM_	*bla*_CTX-M_	*bla*_OXA_
2013 (*n* = 106)	9.4 (4.6, 16.7)	37.7 (28.5, 47.7)	52.8 (42.9, 62.6)	52.8 (42.9, 62.6)
2014 (*n* = 88)	8.0 (3.3, 15.7)	29.5 (20.3, 40.2)	75.0 (64.6, 83.6)	54.5 (43.6, 65.2)
2015 (*n* = 255)	7.5 (4.5, 11.4)	29.8 (24.3, 35.8)	71.4 (65.4, 76.8)	49.8 (43.5, 56.1)
2016 (*n* = 207)	12.6 (8.4, 17.9)	24.6 (18.9, 31.1)	62.8 (55.8, 69.4)	35.7 (29.2, 42.7)
2017 (*n* = 126)	9.5 (5.0, 16.0)	23.8 (16.7, 32.2)	61.1 (52.0, 69.7)	36.5 (28.1, 45.6)
2018 (*n* = 90)	13.3 (7.1, 22.1)	28.9 (19.8, 39.4)	63.3 (52.5, 73.2)	37.8 (27.8, 48.6)

**Table 2:** Linkage analysis summary for DHMMC isolates. The *p*-value for a chi-square test for linkage, the phi coefficient, and the associated *p*-value are presented for each resistance marker pair comparison. The number of isolates for each species is given in parenthesis. An asterisk (*) indicates a statistically significant comparison after the FDR-controlling procedure (q* = 0.025) for both the chi-square test and the phi coefficient.

Species	Markers	Chi-Square *p*-Value	PC	PC *p*-Value
All (*n* = 872)	*bla*_SHV_:*bla*_TEM_	3.75 × 10^−9^ *	0.20	2.74 × 10^−9^ *
	*bla*_SHV_:*bla*_CTX-M_	6.37 × 10^−1^	0.02	6.37 × 10^−1^
	*bla*_SHV_:*bla*_OXA_	1.08 × 10^−1^	0.05	1.08 × 10^−1^
	*bla*_TEM_:*bla*_CTX-M_	1.49 × 10^−3^ *	−0.11	1.46 × 10^−3^ *
	*bla*_TEM_:*bla*_OXA_	3.00 × 10^−6^ *	−0.16	2.68 × 10^−6^ *
	*bla*_CTX-M_:*bla*_OXA_	8.59 × 10^−30^ *	0.38	5.24 × 10^−32^ *
*E. coli* (*n* = 787)	*bla*_SHV_:*bla*_TEM_	5.22 × 10^−1^	0.02	5.23 × 10^−1^
	*bla*_SHV_:*bla*_CTX-M_	2.58 × 10^−1^	−0.04	2.59 × 10^−1^
	*bla*_SHV_:*bla*_OXA_	4.89 × 10^−1^	−0.02	4.89 × 10^−1^
	*bla*_TEM_:*bla*_CTX-M_	1.24 × 10^−6^ *	−0.17	1.07 × 10^−6^ *
	*bla*_TEM_:*bla*_OXA_	1.40 × 10^−9^ *	−0.22	9.50 × 10^−10^ *
	*bla*_CTX-M_:*bla*_OXA_	9.22 × 10^−26^ *	0.37	1.53 × 10^−27^ *
*K. pneumoniae* (*n* = 85)	*bla*_SHV_:*bla*_TEM_	3.36 × 10^−4^ *	0.39	2.34 × 10^−4^ *
	*bla*_SHV_:*bla*_CTX-M_	1.97 × 10^−5^ *	0.46	8.15 × 10^−6^ *
	*bla*_SHV_:*bla*_OXA_	6.84 × 10^−3^ *	0.29	6.44 × 10^−3^ *
	*bla*_TEM_:*bla*_CTX-M_	2.58 × 10^−5^ *	0.46	1.13 × 10^−5^ *
	*bla*_TEM_:*bla*_OXA_	3.84 × 10^−2^	0.22	3.88 × 10^−2^
	*bla*_CTX-M_:*bla*_OXA_	5.69 × 10^−6^ *	0.49	1.72 × 10^−6^ *

**Table 3:** The yearly frequency of *bla*_SHV_, *bla*_TEM_, *bla*_CTX-M_, and *bla*_OXA_ from the U.S. Nationwide Database. Each frequency is presented with a 95% confidence interval. The number of isolates is listed in the first column in parenthesis.

Nationwide U.S.	*bla*_SHV_	*bla*_TEM_	*bla*_CTX-M_	*bla*_OXA_
2013 (*n* = 6)	16.7 (0.4, 64.1)	83.3 (35.9, 99.6)	0.0 (0.0, 45.9)	0.0 (0.0, 45.9)
2014 (*n* = 179)	4.5 (1.9, 8.6)	82.7 (76.3, 87.9)	14.0 (9.2, 19.9)	16.8 (11.6, 23.1)
2015 (*n* = 268)	66.8 (60.8, 72.4)	74.6 (69.0, 79.7)	8.2 (5.2, 12.2)	9.7 (6.4, 13.9)
2016 (*n* = 190)	19.5 (14.1, 25.8)	84.7 (78.8, 89.5)	16.8 (11.8, 22.9)	13.2 (8.7, 18.8)
2017 (*n* = 251)	50.6 (44.2, 56.9)	66.9 (60.7, 72.7)	13.9 (9.9, 18.9)	11.6 (7.9, 16.2)
2018 (*n* = 166)	50.0 (42.2, 57.8)	66.9 (59.2, 74.0)	22.3 (16.2, 29.4)	18.7 (13.1, 25.4)

**Table 4:** Linkage analysis summary for the Nationwide Database Isolates. The *p*-value for a chi-square test for linkage, the phi coefficient, and the associated *p*-value are presented for each resistance marker pair comparison. The number of isolates for each species is given in parenthesis. An asterisk (*) indicates a statistically significant comparison after the FDR-controlling procedure (q* = 0.025) for both the chi-square test and the phi coefficient.

Species	Markers	Chi-Square *p*-Value	PC	PC *p*-Value
All (*n* = 1060)	*bla*_SHV_:*bla*_TEM_	7.03 × 10^−43^ *	−0.42	6.29 × 10^−47^ *
	*bla*_SHV_:*bla*_CTX-M_	2.13 × 10^−1^	−0.04	2.14 × 10^−1^
	*bla*_SHV_:*bla*_OXA_	2.81 × 10^−1^	−0.03	2.81 × 10^−1^
	*bla*_TEM_:*bla*_CTX-M_	1.31 × 10^−9^ *	−0.19	9.80 × 10^−10^ *
	*bla*_TEM_:*bla*_OXA_	3.31 × 10^−17^ *	−0.26	1.02 × 10^−17^ *
	*bla*_CTX-M_:*bla*_OXA_	1.01 × 10^−99^ *	0.65	7.63 × 10^−129^ *
*E. coli* (*n* = 559)	*bla*_SHV_:*bla*_TEM_	2.03 × 10^−6^ *	−0.20	1.68 × 10^−6^ *
	*bla*_SHV_:*bla*_CTX-M_	1.48 × 10^−1^	−0.06	1.49 × 10^−1^
	*bla*_SHV_:*bla*_OXA_	1.52 × 10^−1^	−0.06	1.52 × 10^−1^
	*bla*_TEM_:*bla*_CTX-M_	6.72 × 10^−35^ *	−0.52	2.90 × 10^−40^ *
	*bla*_TEM_:*bla*_OXA_	1.85 × 10^−64^ *	−0.72	2.38 × 10^−89^ *
	*bla*_CTX-M_:*bla*_OXA_	1.83 × 10^−53^ *	0.65	1.05 × 10^−68^ *
*K. pneumoniae* (*n* = 501)	*bla*_SHV_:*bla*_TEM_	6.81 × 10^−10^ *	−0.28	3.45 × 10^−10^ *
	*bla*_SHV_:*bla*_CTX-M_	4.69 × 10^−5^ *	−0.18	4.23 × 10^−5^ *
	*bla*_SHV_:*bla*_OXA_	7.37 × 10^−3^ *	−0.12	7.30 × 10^−3^ *
	*bla*_TEM_:*bla*_CTX-M_	4.73 × 10^−1^	0.03	4.74 × 10^−1^
	*bla*_TEM_:*bla*_OXA_	5.68 × 10^−1^	0.03	5.69 × 10^−1^
	*bla*_CTX-M_:*bla*_OXA_	4.37 × 10^−48^ *	0.65	1.04 × 10^−61^ *

**Table 5:** Percent frequency differences between resistance markers at DHMMC and the Nationwide Database. The frequency of a resistance marker from DHMMC is denoted F_M_, and the frequency of a resistance marker from the National Database is denoted by F_N_. An asterisk (*) indicates a statistically significant comparison after the FDR-controlling procedure (q* = 0.025). The last column provides the 95% confidence interval for the percent difference for a particular resistance marker between the two datasets.

Species	Marker	F*_M_*	F*_N_*	F*_M_—*F*_N_*	*p*-Value	95% CI
All	*bla*_SHV_	9.9	41.0	−31.2	2.79 × 10^−53^ *	(−35.2, −27.2)
	*bla*_TEM_	28.6	74.8	−46.3	1.34 × 10^−91^ *	(−50.7, −41.8)
	*bla*_CTX-M_	65.1	14.2	50.9	2.40 × 10^−117^ *	(46.6, 55.2)
	*bla*_OXA_	44.2	13.3	30.8	6.58 × 10^−52^ *	(26.9, 34.8)
*E. coli*	*bla*_SHV_	2.8	2.5	0.3	7.44 × 10^−1^	(−1.5, 2.0)
	*bla*_TEM_	25.9	89.1	−63.2	1.14 × 10^−115^	(−68.6, −57.8)
	*bla*_CTX-M_	65.8	12.7	53.1	1.75 × 10^−83^	(47.7, 58.5)
	*bla*_OXA_	43.6	12.5	31.1	4.16 × 10^−34^	(26.1, 36.1)
*K. pneumoniae*	*bla*_SHV_	75.3	84.0	−8.7	4.86 × 10^−2^	(−17.4, −0.1)
	*bla*_TEM_	52.9	58.9	−5.9	3.05 × 10^−1^	(−17.3, 5.4)
	*bla*_CTX-M_	58.8	16.0	42.9	1.46 × 10^−18^	(33.3, 52.4)
	*bla*_OXA_	49.4	14.2	35.2	2.65 × 10^−14^	(26.2, 44.3)

**Table 6:** List of primers pairs used to identify *bla*_SHV_, *bla*_TEM_, *bla*_OXA_, and *bla*_CTX-M_ and their expected product size.

Gene	Primer Sequence (5′ to 3′)	Product Size (bp)
*bla*_SHV_	Forward GCCTGTGTATTATCTCCCTGTTAG Reverse TCCCGGCGATTTGCTGATTCC	813
*bla*_TEM_	Forward TGACGCCGGGCAAGAGCA Reverse AAGGGCCGAGCGCAGAAGTG	424
*bla*_OXA_	Forward AGCGCCAGTGCATCAACAG Reverse GCAAAACCCAAACAACAGAAA	300
*bla*_CTX-M_	Forward CGGCCGCGGTGCTGAAGAA Reverse GCTGCCGGTTTTATCCCCCACAA	482

## Data Availability

All data used for this study are available through Supplementary Materials and through the NCBI Isolates Browser database.

## Supplementary Materials

The following are available online at https://www.mdpi.com/article/10.3390/antibiotics10050498/s1. Table S1: Pairwise yearly frequency comparison for blaSHV in the DHMMC database, Table S2: Pairwise yearly frequency comparison for blaTEM in the DHMMC database, Table S3: Pairwise yearly frequency comparison for blaCTX-M in the DHMMC database, Table S4: Pairwise yearly frequency comparison for blaOXA in the DHMMC database, Table S5: Pairwise yearly frequency comparison for blaSHV in the Nationwide database, Table S6: Pairwise yearly frequency comparison for blaTEM in the Nationwide database, Table S7: Pairwise yearly frequency comparison for blaCTX-M in the Nationwide database, Table S8: Pairwise yearly frequency comparison for blaOXA in the Nationwide database, Figure S1: 2% agarose gel of blaCTX-M, blaTEM, and blaOXA PCR products, Figure S2: 2% agarose gel of blaSHV PCR products, Table S9: Number of clinical isolates annually by state.
