# Supplementary material for: Distribution of β-Lactamase Genes in Clinical Isolates from California Central Valley Hospital Deviates from the United States Nationwide Trends
Source: Antibiotics (Basel). 2021 Apr 27;10(5):498. doi: 10.3390/antibiotics10050498 (PMC8146836; doi:10.3390/antibiotics10050498)
Supplement: Supplementary file 1 [file antibiotics-10-00498-s001.zip › antibiotics-1165395-supplementary.pdf]

Supplementary File

Table S1. Pairwise yearly frequency comparison for *bla*SHV in the DHMMC database. The first two columns show the years whose frequencies are being compared. The third column shows the frequency difference, and the last column shows the *p*-value associated with the frequency difference.

Y1	Y2	FY1-FY2	*p*-value
2013	2014	1.5	7.17e-01
2013	2015	2.0	5.28e-01
2013	2016	-3.1	4.12e-01
2013	2017	-0.1	9.81e-01
2013	2018	-3.9	3.89e-01
2014	2015	0.5	8.78e-01
2014	2016	-4.6	2.51e-01
2014	2017	-1.6	6.91e-01
2014	2018	-5.4	2.45e-01
2015	2016	-5.1	6.55e-02
2015	2017	-2.1	4.86e-01
2015	2018	-5.9	9.34e-02
2016	2017	3.0	3.98e-01
2016	2018	-0.8	8.55e-01
2017	2018	-3.8	3.80e-01

Table S2. Pairwise yearly frequency comparison for *bla*TEM in the DHMMC database. The first two columns show the years whose frequencies are being compared. The third column shows the frequency difference, and the last column shows the *p*-value associated with the frequency difference.

Y1	Y2	FY1-FY2	*p*-value
2013	2014	8.2	2.31e-01
2013	2015	7.9	1.42e-01
2013	2016	13.1	1.57e-02
2013	2017	13.9	2.13e-02
2013	2018	8.8	1.92e-01
2014	2015	-0.3	9.64e-01
2014	2016	4.9	3.80e-01
2014	2017	5.7	3.48e-01
2014	2018	0.7	9.23e-01
2015	2016	5.2	2.16e-01
2015	2017	6.0	2.19e-01
2015	2018	0.9	8.70e-01
2016	2017	0.8	8.64e-01
2016	2018	-4.3	4.42e-01
2017	2018	-5.1	4.01e-01

Table S3. Pairwise yearly frequency comparison for *bla*CTX-M in the DHMMC database. The first two columns show the years whose frequencies are being compared. The third column shows the frequency difference, and the last column shows the *p*-value associated with the frequency difference. The asterisk (*) indicate a statistically significant comparison after the False Discovery Rate controlling procedure (*q**=0.025).

Y1	Y2	FY1-FY2	*p*-value
2013	2014	-22.2	1.46e-03*
2013	2015	-18.5	7.11e-04*
2013	2016	-10.0	8.91e-02

2013	2017	-8.3	2.04e-01
2013	2018	-10.5	1.38e-01
2014	2015	3.6	5.12e-01
2014	2016	12.2	4.24e-02
2014	2017	13.9	3.37e-02
2014	2018	11.7	9.21e-02
2015	2016	8.6	5.04e-02
2015	2017	10.3	4.34e-02
2015	2018	8.0	1.55e-01
2016	2017	1.7	7.58e-01
2016	2018	-0.5	9.31e-01
2017	2018	-2.2	7.40e-01

Table S4. Pairwise yearly frequency comparison for *bla*$_{OXA}$ in the DHMMC database. The first two columns show the years whose frequencies are being compared. The third column shows the frequency difference, and the last column shows the *p*-value associated with the frequency difference. The asterisk (*) indicate a statistically significant comparison after the False Discovery Rate controlling procedure (*q**=0.025).

**Y1**	**Y2**	**FY1-FY2**	***p*-value**
2013	2014	-1.7	8.11e-01
2013	2015	3.0	6.00e-01
2013	2016	17.1	3.70e-03*
2013	2017	16.3	1.26e-02
2013	2018	15.1	3.51e-02
2014	2015	4.7	4.43e-01
2014	2016	18.8	2.71e-03*
2014	2017	18.0	8.89e-03*
2014	2018	16.8	2.48e-02
2015	2016	14.1	2.44e-03*
2015	2017	13.3	1.42e-02
2015	2018	12.0	4.93e-02
2016	2017	-0.8	8.89e-01
2016	2018	-2.0	7.38e-01
2017	2018	-1.3	8.49e-01

Table S5. Pairwise yearly frequency comparison for *bla*$_{SHV}$ in the Nationwide database. The first two columns show the years whose frequencies are being compared. The third column shows the frequency difference, and the last column shows the *p*-value associated with the frequency difference. The asterisk (*) indicate a statistically significant comparison after the False Discovery Rate controlling procedure (*q**=0.025).

**Y1**	**Y2**	**FY1-FY2**	***p*-value**
2013	2014	12.2	1.72e-01
2013	2015	-50.1	1.05e-02
2013	2016	-2.8	8.64e-01
2013	2017	-33.9	1.00e-01
2013	2018	-33.3	1.09e-01
2014	2015	-62.3	3.85e-39*
2014	2016	-15.0	1.07e-05*
2014	2017	-46.1	3.00e-24*
2014	2018	-45.5	8.93e-22*
2015	2016	47.3	1.61e-23*
2015	2017	16.2	1.78e-04*

2015	2018	16.8	5.10e-04*
2016	2017	-31.1	2.13e-11*
2016	2018	-30.5	1.21e-09*
2017	2018	0.6	9.05e-01

Table S6. Pairwise yearly frequency comparison for bla_{TEM} in the Nationwide database. The first two columns show the years whose frequencies are being compared. The third column shows the frequency difference, and the last column shows the *p*-value associated with the frequency difference. The asterisk (*) indicate a statistically significant comparison after the False Discovery Rate controlling procedure (q*=0.025).

Y1	Y2	FY1-FY2	*p*-value
2013	2014	0.7	9.67e-01
2013	2015	8.7	6.27e-01
2013	2016	-1.4	9.25e-01
2013	2017	16.4	3.97e-01
2013	2018	16.5	3.98e-01
2014	2015	8.1	4.45e-02
2014	2016	-2.1	5.93e-01
2014	2017	15.7	2.65e-04*
2014	2018	15.8	6.92e-04*
2015	2016	-10.1	9.08e-03*
2015	2017	7.7	5.38e-02
2015	2018	7.8	8.13e-02
2016	2017	17.8	2.10e-05*
2016	2018	17.9	7.45e-05*
2017	2018	0.1	9.89e-01

Table S7. Pairwise yearly frequency comparison for $bla_{CTX\text{-}M}$ in the Nationwide database. The first two columns show the years whose frequencies are being compared. The third column shows the frequency difference, and the last column shows the *p*-value associated with the frequency difference. The asterisk (*) indicate a statistically significant comparison after the False Discovery Rate controlling procedure (q*=0.025).

Y1	Y2	FY1-FY2	*p*-value
2013	2014	-14.0	3.25e-01
2013	2015	-8.2	4.64e-01
2013	2016	-16.8	2.72e-01
2013	2017	-13.9	3.25e-01
2013	2018	-22.3	1.92e-01
2014	2015	5.8	5.18e-02
2014	2016	-2.9	4.45e-01
2014	2017	0.0	9.95e-01
2014	2018	-8.3	4.43e-02
2015	2016	-8.6	4.76e-03*
2015	2017	-5.7	3.68e-02
2015	2018	-14.1	3.19e-05*
2016	2017	2.9	4.01e-01
2016	2018	-5.4	1.95e-01
2017	2018	-8.3	2.73e-02

Table S8. Pairwise yearly frequency comparison for bla_{OXA} in the Nationwide database. The first two columns show the years whose frequencies are being compared. The third column shows the frequency difference, and the last column shows the *p*-value associated with the frequency

difference. The asterisk (*) indicate a statistically significant comparison after the False Discovery Rate controlling procedure (q^*=0.025).

Y1	Y2	FY1-FY2	*p*-value
2013	2014	-16.8	2.73e-01
2013	2015	-9.7	4.23e-01
2013	2016	-13.2	3.41e-01
2013	2017	-11.6	3.77e-01
2013	2018	-18.7	2.42e-01
2014	2015	7.1	2.72e-02
2014	2016	3.6	3.32e-01
2014	2017	5.2	1.22e-01
2014	2018	-1.9	6.41e-01
2015	2016	-3.5	2.47e-01
2015	2017	-1.9	4.93e-01
2015	2018	-9.0	7.15e-03*
2016	2017	1.6	6.11e-01
2016	2018	-5.5	1.54e-01
2017	2018	-7.1	4.26e-02

Table S9. The Number of Clinical Isolates Each Year by State from the US Nationwide Database. N/A are clinical isolates with no location information.

Year	States (Number of Isolates)
2013 (6)	Missouri (4), Washington (2)
2014 (179)	Washington (104), Pacific Northwest (43), Massachusetts (13), California (6), N/A (3), Texas (3), North Carolina (2), Illinois (1), Iowa (1), Maryland (1), Midwest (1), Oklahoma (1)
2015 (268)	N/A (157), Washington (69), California (19), Illinois (5), Minnesota (5), Virginia (5), Missouri (2), Connecticut (1), Kansas (1), Maryland (1), Massachusetts (1), Michigan (1), Oklahoma (1)
2016 (190)	N/A (156), California (9), Texas (9), Minnesota (6), Virginia (5), Colorado (1), Massachusetts (1), Mississippi (1), New York (1), Pennsylvania (1)
2017 (251)	N/A (93), Massachusetts (51), Washington (26), New York (17), California (16), Pennsylvania (9), Minnesota (8), Missouri (8), Illinois (5), Texas (4), Connecticut (2), North Carolina (2), Puerto Rico (2), Colorado (1), Florida (1), Michigan (1), Mississippi (1), Montana (1), Nevada (1), Ohio (1), Virginia (1)
2018 (166)	Missouri (45), Minnesota (37), Pennsylvania (33), California (23), Illinois (8), Maryland (5), Virginia (5), N/A (3), Wisconsin (2), Georgia (1), Massachusetts (1), New York (1), Tennessee (1), Texas (1)

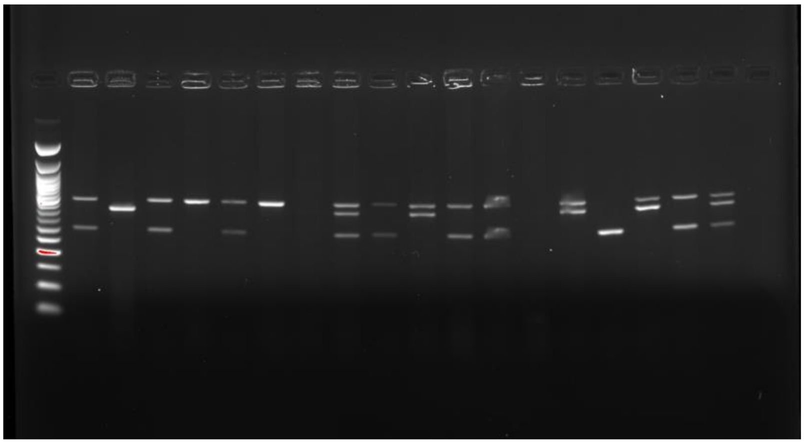

Figure S1. 2% agarose gel of *bla*$_{\text{CTX-M}}$, *bla*$_{\text{TEM}}$, and *bla*$_{\text{OXA}}$ PCR products**.** In lanes 2-18, isolates 337-372. Lane 19 is the positive control, isolate 389, and shows 3 bands at 482bp for *bla*$_{\text{CTX-M}}$, 424bp for *bla*$_{\text{TEM}}$, and 300bp for *bla*$_{\text{OXA}}$. Lane 1 1kb NEB Ladder; Lane 2: 356, *E.coli* isolate positive for *bla*$_{\text{CTXM}}$ and *bla*$_{\text{OXA;}}$ Lane 3: 357, *E.coli* isolate positive for *bla*$_{\text{TEM;}}$ Lane 4: 358, *E.coli* isolate positive for *bla*$_{\text{CTXM}}$ and *bla*$_{\text{OXA;}}$ Lane 5: 359, *E.coli* isolate positive for *bla*$_{\text{CTXM;}}$ Lane 6: 360, *E.coli* isolate positive for *bla*$_{\text{CTXM}}$ and *bla*$_{\text{OXA;}}$ Lane 7: 36, *E.coli* isolate positive for *bla*$_{\text{CTXM}}$; Lane 8: 362, *E.coli* isolate negative for *bla*$_{\text{CTX-M}}$, *bla*$_{\text{TEM}}$, and *bla*$_{\text{OXA}}$; Lane 9: 363, *E.coli* isolate positive for *bla*$_{\text{CTXM}}$, *bla*$_{\text{TEM}}$ and *bla*$_{\text{OXA}}$; Lane 10: 364, *E.coli* isolate positive for *bla*$_{\text{CTXM}}$ and *bla*$_{\text{OXA}}$; Lane 11: 365, *E.coli* isolate positive for *bla*$_{\text{CTXM}}$ and *bla*$_{\text{TEM}}$; Lane 12: 366, *E.coli* isolate positive for *bla*$_{\text{CTXM}}$ and *bla*$_{\text{OXA}}$; Lane 13: 367, *E.coli* isolate positive for *bla*$_{\text{CTXM}}$ and *bla*$_{\text{OXA}}$; Lane 14: 368, *E.coli* isolate negative for *bla*$_{\text{CTX-M}}$, *bla*$_{\text{TEM}}$, and *bla*$_{\text{OXA;}}$ Lane 15: 369, *K. pneumoniae* isolate positive for *bla*$_{\text{CTXM}}$ and *bla*$_{\text{TEM}}$; Lane 16: 370, *E.coli* isolate positive for *bla*$_{\text{OXA}}$; Lane 17: 371, *E.coli* isolate positive for *bla*$_{\text{CTXM}}$ and *bla*$_{\text{TEM}}$; *E.coli* isolate: Lane 19: Positive PCR control 389, *K. pneumoniae* isolate positive for *bla*$_{\text{CTX-M}}$, *bla*$_{\text{TEM}}$, and *bla*$_{\text{OXA}}$; Lane 20: Negative PCR control, water.

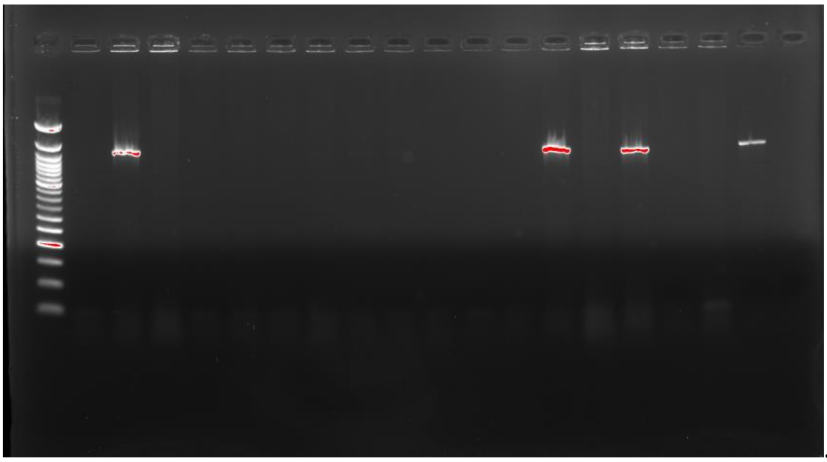

Figure S2. 2% agarose gel of *bla*$_{\text{SHV}}$ PCR products. In lanes 2-18, DHMMC isolates 561-564, 567-579. Lane 19 is the positive control, isolate 389, and shows a band at 813bp for *bla*$_{\text{SHV}}$. Lane 1 1kb NEB Ladder; Lane 2: 561, *E.coli* isolate negative for *bla*$_{\text{SHV}}$; Lane 3: 562, *E.coli* isolate positive for *bla*$_{\text{SHV}}$; Lane 4: 563, *E.coli* isolate negative for *bla*$_{\text{SHV}}$; Lane 5: 564, *E.coli* isolate negative for *bla*$_{\text{SHV}}$; Lane 6: 567, *E.coli* isolate negative for *bla*$_{\text{SHV}}$; Lane 7: 568, *E.coli* isolate negative for *bla*$_{\text{SHV}}$; Lane 8: 569, *E.coli* isolate negative for *bla*$_{\text{SHV}}$; Lane 9: 570, *E.coli* isolate negative for *bla*$_{\text{SHV}}$; Lane 10: 571, *E.coli* isolate negative for *bla*$_{\text{SHV}}$; Lane 11: 572, *E.coli* isolate negative for *bla*$_{\text{SHV}}$; Lane 12: 573, *E.coli* isolate negative for *bla*$_{\text{SHV}}$; Lane 13: 574, *E.coli* isolate negative for *bla*$_{\text{SHV}}$; Lane 14: 575, *K. pneumoniae* isolate positive for *bla*$_{\text{SHV}}$; Lane 15: 576, *E.coli* isolate negative for *bla*$_{\text{SHV}}$; Lane 16: 577, *K. pneumoniae* positive for *bla*$_{\text{SHV}}$; Lane 17: 578, *E.coli* isolate negative for *bla*$_{\text{SHV}}$; Lane 18: 579, *E.coli* isolate negative for *bla*$_{\text{SHV}}$; Lane 19: Positive PCR control 389, *K. pneumoniae* isolate positive for *bla*$_{\text{SHV}}$; Lane 20: Negative PCR control, water.
